# Stone heat treatment in the Early Mesolithic of southwestern Germany: Interpretation and identification

**DOI:** 10.1371/journal.pone.0188576

**Published:** 2017-12-06

**Authors:** Patrick Schmidt, Océane Spinelli Sanchez, Claus-Joachim Kind

**Affiliations:** 1 Department of Early Prehistory and Quaternary Ecology, Eberhard Karls University of Tübingen, Tübingen, Germany; 2 Department of Geosciences, Applied Mineralogy, Eberhard Karls University of Tübingen, Tübingen, Germany; 3 Maison de l’archéologie, IRAMAT-CRP2A UMR 5060, Université Bordeaux Montaigne, Pessac, France; 4 State Office for Cultural Heritage Baden-Württemberg, Esslingen am Neckar, Germany; New York State Museum, UNITED STATES

## Abstract

The Early Mesolithic of southwestern Germany, the so-called Beuronian (9600–7100 BC), is a period of important transformations in the way people lived, in their subsistence and in the stone tools they produced. One of the perhaps most spectacular re-inventions of that time is heat treatment of stones prior to their manufacture into tools. Although heat treatment has been understood as one of the defining characteristics of the Beuronian of southwestern Germany, and although its existence has been known for almost 30 years now, relatively few systematic studies on it are available. In this paper, we present such a study, aiming to shed light on two questions: (1) what technique and heating parameters were used in the Beuronian and (2) how reliable are the macroscopic proxies traditionally used to identify heat treatment in this context? We investigate these questions using a non-destructive archaeometric technique for measuring past heating temperatures of heat-treated stones and a quantitative surface roughness analysis aiming to understand the relations between surface aspect and heat treatment. These methods are applied to 46 Jurassic chert artefacts from the site Helga-Abri located in the Swabian Alb region of southwestern Germany. Our results document that an opportunistic low-investment procedure was used to heat stone, probably relying on the use of the above-ground part of regular camp-fires. We also found that the traditionally used macroscopic criteria, such as colour and surface gloss, cannot be unambiguously used to identify heat treatment in assemblages made from Jurassic chert. These findings have important implications for our understanding of the Beuronian lithic chaîne opératoire in terms of the investment in time and resources necessary, and for the refinement of archaeological techniques used to identify heat treatment in the Mesolithic of the Swabian Alb.

## Introduction

The Swabian Alb or Swabian Jura is a ~200 km long and ~70 km wide lime stone plateau of Jurassic (201–145 Ma) age in southwestern Germany. The region has recently become world famous because of its Upper Palaeolithic cave sites [1–3] that have yielded some of the earliest manifestations of figurative art and musical instruments (see for example [4–6]). The Swabian Alb is, however, also home to more than 20 Mesolithic cave and rock-shelter sites [7] and it therefore played an important role in the early research on this period in southwestern Germany. This research really started in the beginning of the 20^th^ century, when the famous skull burials of Ofnet Cave [8] were excavated in 1908. Intensive research was conducted at several cave, rock-shelter [9] and open air [10] sites in the 1920s and 30s, mainly to investigate the chronological framework of the German Mesolithic. About 40 years later, W. Taute was the first to show the typological and chronological divisions of the German Mesolithic [11]. In the following period, from the 1990s till today, Mesolithic archaeology in Germany began to focus on new questions about land use, the exploitation of resources [12, 13], stone raw material exploitation and the technology of stone knapping [14–17].

Typical lithic artefact for the Mesolithic are so called microliths. These are small triangular or rectangular stone tools with a length of only one to three centimeters. They were used as implements that were hafted to a wooden projectile. Taute’s typological chronology of Mesolithic southwestern Germany begins with the so-called “Beuronian” (9600–7100 Cal BC) and ends with the “Late Mesolithic” (7100–4500 Cal BC) as the final stage [11]. The Beuronian is further subdivided into Beuronian A, B and C, of which the earlier phases A and B are characterized by larger triangles with differing angles and by triangular points with dorso-ventrally retouched bases. The Beuronian C is mainly characterized by extremely scalene triangles. The Late Mesolithic is characterized by rectangular microliths such as trapezes and a new technique for producing regular blades. Another trait characterising the earlier Mesolithic is heat treatment. Hahn [14] noted that during the Beuronian, local chert often was treated with fire before knapping, to optimize its fracture qualities. Following this observation, several other works explored Beuronian heat treatment, providing first insights into its relative prevalence in different Beuronian assemblages [18] and investigating possibly applied heating techniques experimentally [19]. However, many aspects of Mesolithic heat treatment are still poorly understood: the technique used for heating, the parameters applied during heating, the timing of heat treatment in the reduction series or the methodological pathway for identifying heat treatment in Beuronian assemblages. In this paper, we consider two of these questions:

1. What technique was used for heat treatment in the Beuronian of the Swabian Alb? Was it a time- and resource-intensive process or was it a fast procedure that may be performed along other daily activities? Either of these two possibilities would have important implications for our perception of the Beuronian lithic chaîne opératoire.
2. How can heat treatment be recognised macroscopically. Many previous works have used criteria such as colour or surface gloss to estimate whether Mesolithic artefacts from the Swabian Alb were heated or worked untransformed. However, no methodological studies on the reliability of these macroscopic criteria are available

To investigate question (1), we apply a method for estimating the heating temperatures of heat-treated artefacts [20] to a selection of lithic implements from the site Helga-Abri located on the Swabian Alb, hoping in this way to gain information about the technique and procedure that were used to heat stones to these temperatures. Question (2) is approached with a quantitative analysis of the surface roughness on artefact removal scars that provides data on the relationship of surface gloss and heat treatment.

## Methods and materials

### The site and its stratigraphy

Helga-Abri [21] is a small rock-shelter located a few meters uphill on the southern flank of the limestone cliff that also houses the Palaeolithic site Hohle Fels [4, 22]. Helga-Abri has yielded a cultural sequence dating from the late Magdalenian to the early Mesolithic. The site was excavated several times, with J. Hahn’s campaigns between 1977 and 1984 having yielded the most complete stratigraphic sequence: six Mesolithic layers, numbered from IIF1 to IIF6, date to the Beuronian B to C (IIF1: possibly C; IIF2: C; IIF3: possibly C; IIF4 possibly B; IIF5 and 6: B)[21]. Magdalenian (17000–12000 Cal BP) deposits (not considered in this work) were found at the base of a hiatus below these six Mesolithic layers. The Helga-Abri sequence documents several innovations (bow-and-arrow; domestication of dogs) and provides a window onto the rapidly changing environmental conditions of the terminal Pleistocene and early Holocene [23]. The site’s lithic industry and raw material provenance patterns have recently been the subject of a doctoral thesis at Tübingen University, that may provide detailed insight into these aspects to the interested reader [24].

### Archaeological samples

**Fig 1:**
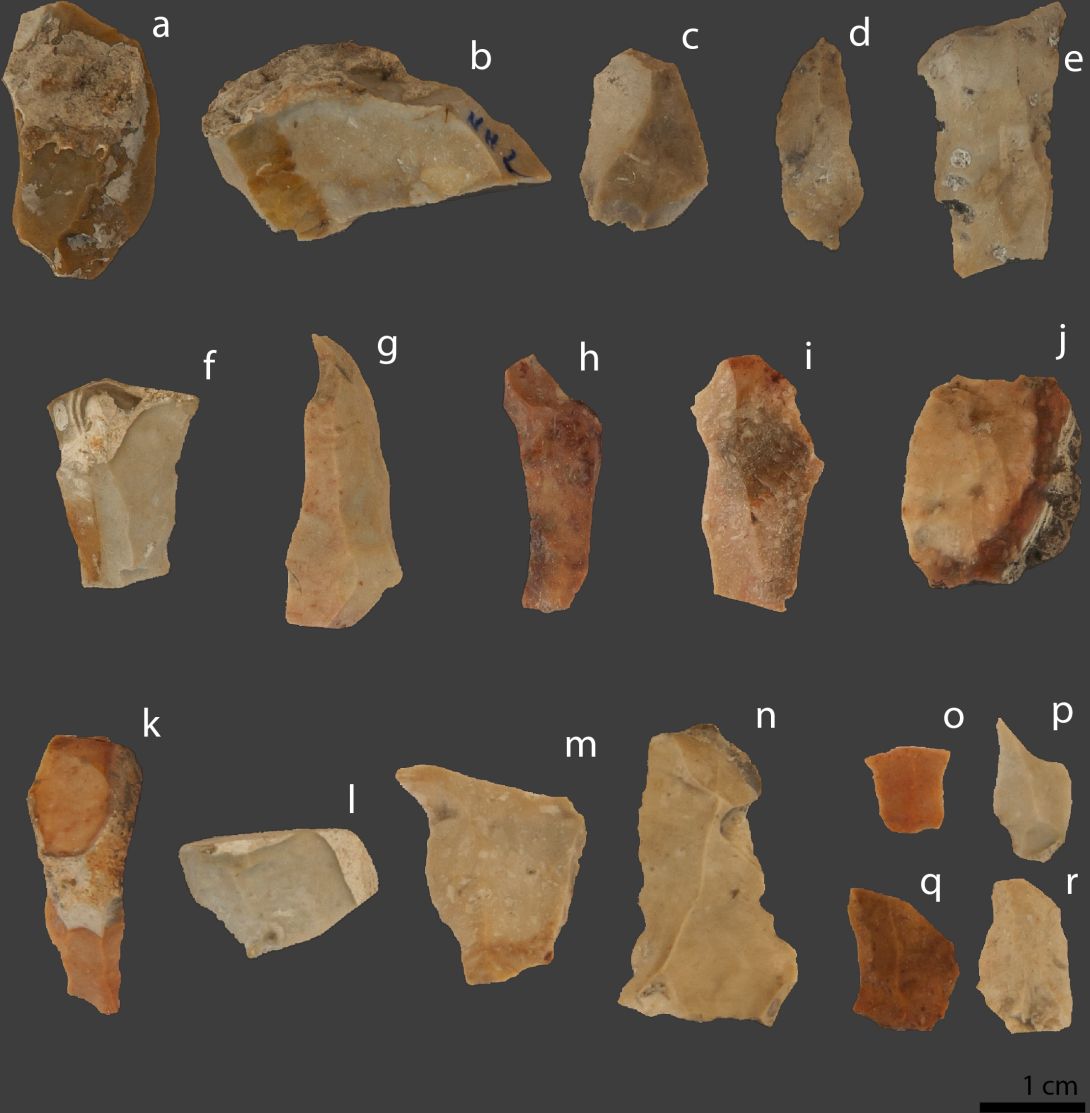
Photographs of a selection of analysed Helga-Abri artefacts. Pieces in the top row were assigned to the group *Not-heated*. a: No accession N° (7); b: No accession N° (8); c: HA10-IIF3b-85 (2): d: HA10a-IIF2-47-2-471 (1); e: HA11-IIF2-30 (3). Brackets after the artefacts’ accession numbers are the short numbers of Table 1. Pieces in the middle row were assigned to the group *Gloss contrast*. f: HA20a-IIF5-109 (17); g: HA10a-IIF2-32a2 (14); h: HA11-IIF1-27 (16); i: HA11c-IIF3b-112 (15); j: HA21-IIF3b-42 (19). Pieces in the bottom row were assigned to the *Test* group. k: HA11d-IIF3-76 (37); l: HA20b-IIF6-133 (40); m: HA10-IIF3-41 (33); n: HA10-IIF3-87 (32); o: HA11d-IIF4-92 (34); p: HA20a-IIF6-127 (39); q: HA21-IIF3b-51 (41); r: HA10-IIF1-12 (3).

**Fig 2:**
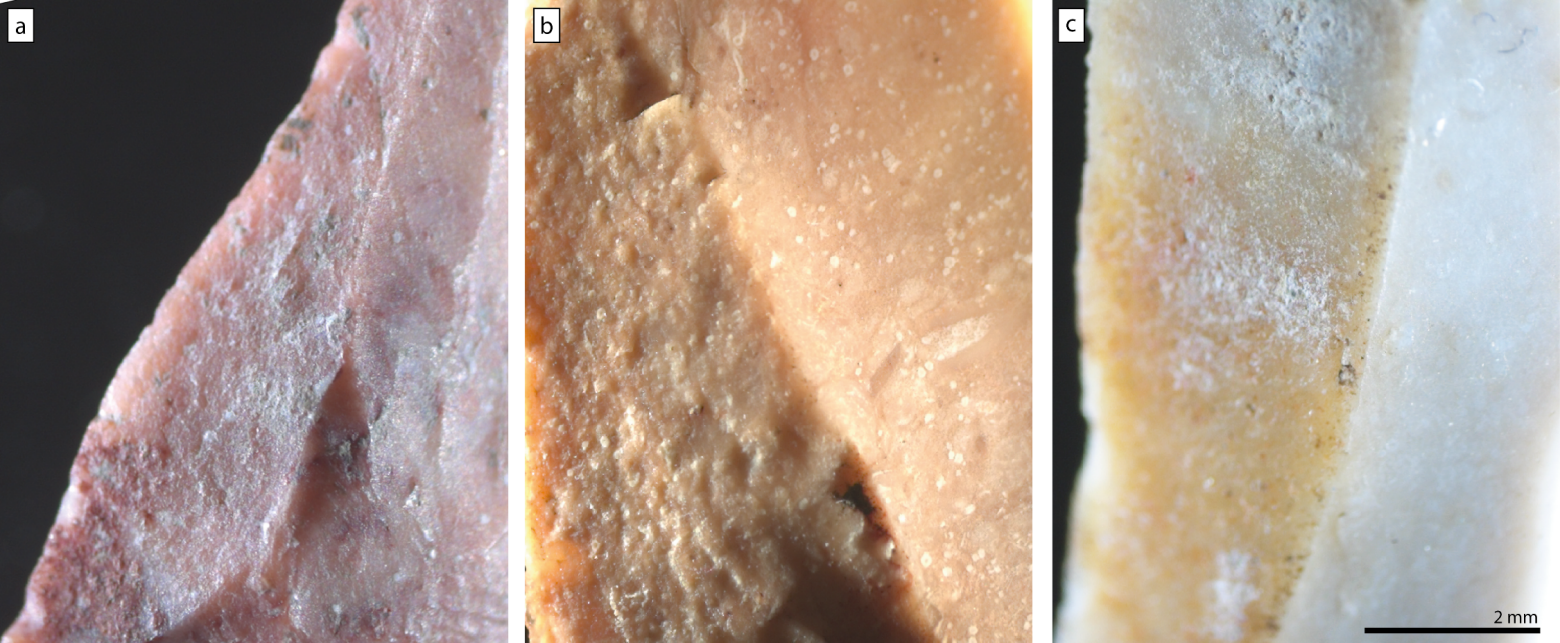
Close-up photos of gloss contrast on three Helga-Abri artefacts. a: HA11-IIF1-27 (16), note the rough appearance of the removal scar in the centre of the picture compared to the adjacent negatives on the right. b: HA11c-IIF3b-112 (15), note the rough appearance of the left removal scar compared to the adjacent scar on its right. c: HA20a-IIF5-109 (17), note the rough appearance of the left removal scar compared to the adjacent scar on its right. Brackets after the artefacts’ accession numbers are the short numbers of Table 1.

Forty-six (46) archaeological samples, made from local Jurassic chert, were selected for analysis from the Mesolithic Helga-Abri collection. Because of the sparsity of artefacts well suited for our infrared analysis (artefacts must contain large enough cortex-free zones that are also thin enough to allow IR transmission), the samples were randomly chosen from all six Mesolithic layers IIF1 to IIF6 (the only criterion for section was the suitability for analysis). Some of these 46 artefacts are shown in Fig 1. In a first step, these samples were inspected for macroscopic heat treatment proxies. Based on these proxies, the following three groups were made: (1) Artefacts assigned to the group *Not-heated* (n = 11) were selected because they either showed patches or zones of bright yellow colour or because they showed weak overall gloss intensity on their removal scars. Bright yellow colour can be used as criterion to identify unheated chert because the accessory minerals causing this colour would most likely have turned red or reddish upon heating (if these minerals are iron-rich and undergo oxidation). Overall gloss intensity is a qualitative estimation of the overall magnitude of the surface lustre of all removal scars on an artefact. The identification of weak, intermediate or strong surface gloss on an artefact may allow to estimate whether it was knapped after heat treatment or not (for the mechanisms involved see [25], for identifying heat treatment on artefacts see [26–29]). (2) Artefacts assigned to the group *Gloss contrast* (n = 8) show the coexistent presence of matt pre- and shinier post-heating removal scars. The presence of removal scars from before and after heat treatment is the most secure macroscopic criterion for identifying heat treatment of stone artefacts because it documents the sequence of pre-heat treatment knapping, subsequent modification of the chert’s fracture mechanics and a second stage of post-heat treatment knapping. Examples of gloss contrast are shown in Fig 2. (3) Artefacts assigned to the *Test* group (n = 27) do not show gloss contrast, i.e. they are not diagnostic heat-treated artefacts, but their overall gloss intensity is intermediate to strong or they are slightly to intensively red. In traditional archaeological estimations of heat-treated vs. not-heated pieces, the artefacts in our *Test* group would likely have been identified as heat-treated. When choosing these artefacts, special attention was payed not to include artefacts with signs of overheating (pot-lids, crazing, internal fracturing [30]). Overheated artefacts may have been subjected to post-depositional burning at high temperatures, making it impossible to estimate heating temperatures of intentional heat treatment. Sample numbers, dimensions and the artefacts’ assignment to the three groups are summarised in Table 1.

**Table 1:** Samples, sample dimensions and their correspondence with short sample numbers.

Macroscopic Group	Short sample N°	Accession N°	Length (mm)	Breadth (mm)	Thickness (mm)	Mass (g)
**Not-heated**	1	HA10a-IIF2-47-2-471	0.8	2.1	0.3	0.4
**Not-heated**	2	HA10-IIF3b-85	1.3	1.7	0.4	0.9
**Not-heated**	3	HA11-IIF2-30	2.6	1.5	0.5	1.6
**Not-heated**	4	HA20a-IIF3-9	1.4	0.7	0.3	0.2
**Not-heated**	5	HA21-IIF3b-58	1.9	1.9	0.5	1.7
**Not-heated**	6	HA21-IIF4-80	1.4	0.7	0.1	0.1
**Not-heated**	7	No accession N°	2.8	1.5	0.5	2
**Not-heated**	8	No accession N°	3.5	2	1	5.2
**Not-heated**	9	v65-31-17-HA	1.5	0.8	0.2	0.3
**Not-heated**	10	HA-20-IIF4-31	1.2	0.9	0.3	0.2
**Not-heated**	11	HA-21-IIF4-131	2.3	1	0.3	0.7
**Gloss contrast**	12	HA0a-IIF2-15	3	1.1	0.3	1
**Gloss contrast**	13	HA20a-IIF5-59	3.1	1.3	0.6	1.3
**Gloss contrast**	14	HA10a-IIF2-32a2	1.1	2.8	0.4	1
**Gloss contrast**	15	HA11c-IIF3-112	1	2.3	0.2	0.5
**Gloss contrast**	16	HA11-IIF1-27	0.9	2.5	0.4	0.8
**Gloss contrast**	17	HA20a-IIF5-109	1.4	1.9	0.4	1.2
**Gloss contrast**	18	HA21-185.1	1.1	1.1	0.2	0.1
**Gloss contrast**	19	HA21-IIF3b-42	1.6	2.1	0.6	1.9
**Test**	20	HA21-IIF3b-71	1.4	1.9	0.4	1.1
**Test**	21	HA20a-IIF6-122	2	0.8	0.3	0.3
**Test**	22	HA21-IIF2-5	2.4	0.9	0.2	0.7
**Test**	23	HA21-IIF2-Z5	1.4	0.8	0.2	0.1
**Test**	24	HA21-IIF3b-53	1.5	1	0.3	0.3
**Test**	25	HA21-IIF3-34	1.2	1	0.3	0.3
**Test**	26	HA21-IIF3b-37	1.2	1.1	0.1	0.2
**Test**	27	HA20a-IIF6-118-1	1	2.3	0.3	0.6
**Test**	28	HA20-IIF2	1.1	1.6	0.2	0.3
**Test**	29	HA11-IIF3-37	2.3	2.6	0.6	3.8
**Test**	30	HA10-IIF1-12	0.9	1.5	0.2	0.3
**Test**	31	HA10-IIF2-24	1.1	1.9	0.3	0.5
**Test**	32	HA10-IIF3-87	1.5	2.9	0.5	2.6
**Test**	33	HA10-IIF3-41	1.6	2.3	0.3	1.2
**Test**	34	HA11d-IIF4-92	0.8	0.8	0.1	0.1
**Test**	35	HA11c-IIF3-56	0.3	1.4	0.4	0.5
**Test**	36	HA11d-IIF1-92	1.7	2.9	0.4	1.7
**Test**	37	HA11d-IIF3-76	1.1	2.5	0.2	0.5
**Test**	38	HA11-IIF2-31	1	1.1	0.2	0.2
**Test**	39	HA20a-IIF6-127	0.8	1.4	0.2	0.1
**Test**	40	HA20b-IIF6-133	1.2	1.8	0.5	1.1
**Test**	41	HA21-IIF3b-51	1	1.4	0.3	0.4
**Test**	42	HA21-IIF4-115	1.2	1.6	0.3	0.7
**Test**	43	HA20b-IIF4-71	2	1.7	0.5	1.6
**Test**	44	HA20b-IIF5-83	2.9	1.2	0.3	1
**Test**	45	HA20-IIF4-48	1.9	2.6	0.4	2.1
**Test**	46	HA21-IIF3b-68	1.3	1.9	0.3	0.8

Entries in the column ‘Macroscopic group’ assign each artefact to the three groups initially made during sample selection. In all following figures and tables, artefacts are only plotted with their short sample numbers. Length measurements are not axial lengths but correspond to the maximum dimension of the artefacts. Breadths were measured perpendicularly to the lengths. Thickness was measured at the thickest spot of each artefact.

### Geological samples

A set of reference samples of the same Jurassic chert, that had clearly never been heated, was collected in the surroundings of Helga-Abri. No permissions were required for collecting the rock samples used for this study as chert is not a rare or precious resource in Germany. The land owners of all sampling locations gave permission to collect samples on their land. No other permissions were required for conduction the study and the study did not involve endangered or protected species. To establish a ‘calibration series’ for the determination of the 46 artefacts’ heating temperatures, we also experimentally heat-treated one of these reference samples at different temperatures. As no detailed studies on the raw materials used in Helga-Abri are available, we collected samples in secondary position from ploughed fields; sampling locations were chosen as a function of their proximity to Helga-Abri and their accessibility. At both these locations individual nodules vary in size, ranging from 1 to 10 cm. Most chert is broken there, due to intensive ploughing, but some nodules can still be found intact showing white cortex all round. We collected three of these larger intact nodules with cortex as reference material. Samples SJ-17-02b and c were collected from a field at N48°25'28.8'' E9°47'27'', approximately 5 km to the north-east of Helga-Abri, and sample SJ-17-05a was collected at 48°26'7.92'' 9°50'0.26'', approximately 8 km to the north-east of the site. All three samples are whitish and show a dull surface aspect on fresh fracture surfaces. Both sampling locations were previously identified as potential raw material sources for several nearby Palaeolithic and Mesolithic archaeological sites [31] and spot SJ-17-05 lies directly beside a local, locally known as “Borgerhau” Forest, that was extensively quarried for Jurassic chert in the local Neolithic [32]. The three samples were further sectioned by knapping to produce 17 flakes, 10 of which were analysed unheated (samples were only heated to 110°C for analytical purposes, see below) and 7 of which were experimentally heat-treated and compared with the 46 archaeological samples. Sample numbers and experimental heating temperatures are summarised in Table 2.

**Table 2:** Infrared 4545/4469 cm^-1^ absorbance ratio values of geological and experimental sample flakes.

Experiment	Sample N°	Temperature (°C)	IR Hydration Ratio
**Geological sample**	SJ-17-05a	110	1.030 ±0.04
**Geological sample**	SJ-17-05a	110	1.004 ±0.04
**Geological sample**	SJ-17-02b	110	1.057 ±0.04
**Geological sample**	SJ-17-02b	110	1.015 ±0.04
**Geological sample**	SJ-17-02b	110	1.053 ±0.04
**Geological sample**	SJ-17-02b	110	1.033 ±0.04
**Geological sample**	SJ-17-02b	110	1.064 ±0.04
**Geological sample**	SJ-17-02c	110	1.004 ±0.04
**Geological sample**	SJ-17-02c	110	1.002 ±0.04
**Geological sample/ Experimental HT**	SJ-17-02c	110	1.054 ±0.04/8
**Experimental HT**	SJ-17-02c	200	1.086 ±0.08
**Experimental HT**	SJ-17-02c	250	1.162 ±0.08
**Experimental HT**	SJ-17-02c	300	1.187 ±0.08
**Experimental HT**	SJ-17-02c	350	1.240 ±0.08
**Experimental HT**	SJ-17-02c	400	1.356 ±0.08
**Experimental HT**	SJ-17-02c	450	1.506 ±0.08
**Experimental HT**	SJ-17-02c	500	1.926 ±0.08

‘Geological sample’ = direct measurement of the sample without heat treatment. ‘Experimental HT’ = different flakes of a single sample are experimentally heat-treated at different temperatures. ‘Geological sample’ were all heated to 110°C for analytical reasons: the complete evaporation of interstitial water before controlled saturation of the pore-space with deionised H_2_O.

### Methods and experimental setup

Archaeometric analyses of the artefacts’ heating temperatures were conducted using infrared (IR) light transmission through the artefacts. The theoretical background and detailed experimental setup of the analyses are explained in Schmidt et al. [20] and only the information absolutely necessary for understanding the method are repeated here. The analyses rely on the measurement of the transmission of near IR radiation, directly through lithic artefacts (zones of remaining cortex and patination should be avoided). The non-destructive measurements result in an IR absorption spectrum between 4000 and 4800 cm^-1^ that contains an absorption band caused by SiOH. The shape of this absorption band (measured as the ratio between the linear absorbances at 4545 cm^-1^ and 4469 cm^-1^, short notation of the ratio: 4545/4469 cm^-1^) is partly influenced by the quantity of water held in the open pore-space of the samples. The mechanism behind this is the chemical interaction of this pore-water with surface SiOH (hydrogen bonding). More pore-water causes a shift to lower frequencies, less pore-water causes a relatively larger band-component at higher wavenumbers [33]. The band’s shape is therefore an indirect measure of the quantity of water in open pores and, if all available pore-space is completely filled with water, also of the volume of open pore-space of the sample itself. When chert is heat-treated, it gradually loses such open pore-space [25, 34, 35]. Schmidt et al.’s [20] method aims at detecting past heating through the measurement of a sample’s pore-space with respect to the pore-space of another sample of the same rock type that was not heated. Not-heated reference samples ideally come from the same find layer in the analysed site (internal reference) because they were subjected to identical taphonomic processes. This is important because some taphonomic processes were found to alter the hydroxylation of chert [36], consequently also influencing the measured IR signal. If such an internal reference is not available, or if it cannot be established with certainty that the internal reference is unheated, an external reference made from the same material may be used (see for example [37]). It must however be kept in mind that in this case, the 4545/4469 cm^-1^ ratio value obtained from the external reference can be slightly different than the ratio values of not-heated artefacts because both groups of materials were subjected to different taphonomic processes, limiting somehow the significance of the study.

Both samples compared in this way, the one tested for past heating and the reference, must undergo an identical protocol, allowing for total filling of their open pore-space with deionized or distilled H_2_O. A higher value of the 4545/4469 cm^-1^ ratio in the tested sample, as compared with that ratio of a not-heated reference sample, indicates that the former was subjected to heating in the past. Heating temperature can be estimated by combining these measurements with measurements of experimentally heat-treated reference samples of the same rock. Subsamples of a reference sample are heated to different temperatures, rehydrated with the same protocol, and then analysed for their 4545/4469 cm^-1^ ratio values after different temperature steps. The comparison between the ratio values of archaeological samples and the ratio of the reference allows to estimate the temperature range the archaeological sample was heated to.

To apply this method to our Helga-Abri samples, all archaeological pieces were dried at 110°C for 28 hours to dehydrate their open pore space and then rehydrated in deionized H_2_O for 48h at room temperature and ambient pressure. Geological reference samples were treated along with the 46 archaeological samples, applying the identical protocol. One of the 3 reference samples underwent experimental heat treatment to estimate the heating temperature of the archaeological samples. For this seven supplementary flakes were knapped from this sample and each was heated to another temperature in an electrical furnace (200°C, 250°C, 300°C, 350°C, 400°C, 450°C and 500°C) with a heating rate of ~20°/h (and a dwell time of 2h, as justified by the data in [38]). After each temperature step, the samples were cooled to room temperature overnight to avoid fracturing induced by excessively fast cooling and then rehydrated in deionized H_2_O for 48h at room temperature and ambient pressure to saturate their open pore space with water. No fracturing of the sample was observed with this protocol.

In a second step, the surface roughness of 42 of the Helga-Abri artefacts was measured with a Laser Scanning Microscope (LSM) (four of the artefacts only showed surfaces that were either too concave, not large enough or that contained topographic features that made analysis with a LSM impossible). LSM analyses were performed because several works (see for example [27, 28, 39, 40]) found that heat treatment modifies the fracture pattern of silica rocks, allowing smoother surface removals after the treatment. Samples previously assigned to the group *Gloss contrast* were measured on both types of removal scars (pre-HT and post-HT) where this was possible. On four of these *Gloss contrast* artefacts it was not possible to measure both types of removal scars (same reasons as explained above). The surface roughness values obtained in this way were then compared with the IR spectroscopic analysis.

### Analytical equipment and experimental error

**Fig 3:**
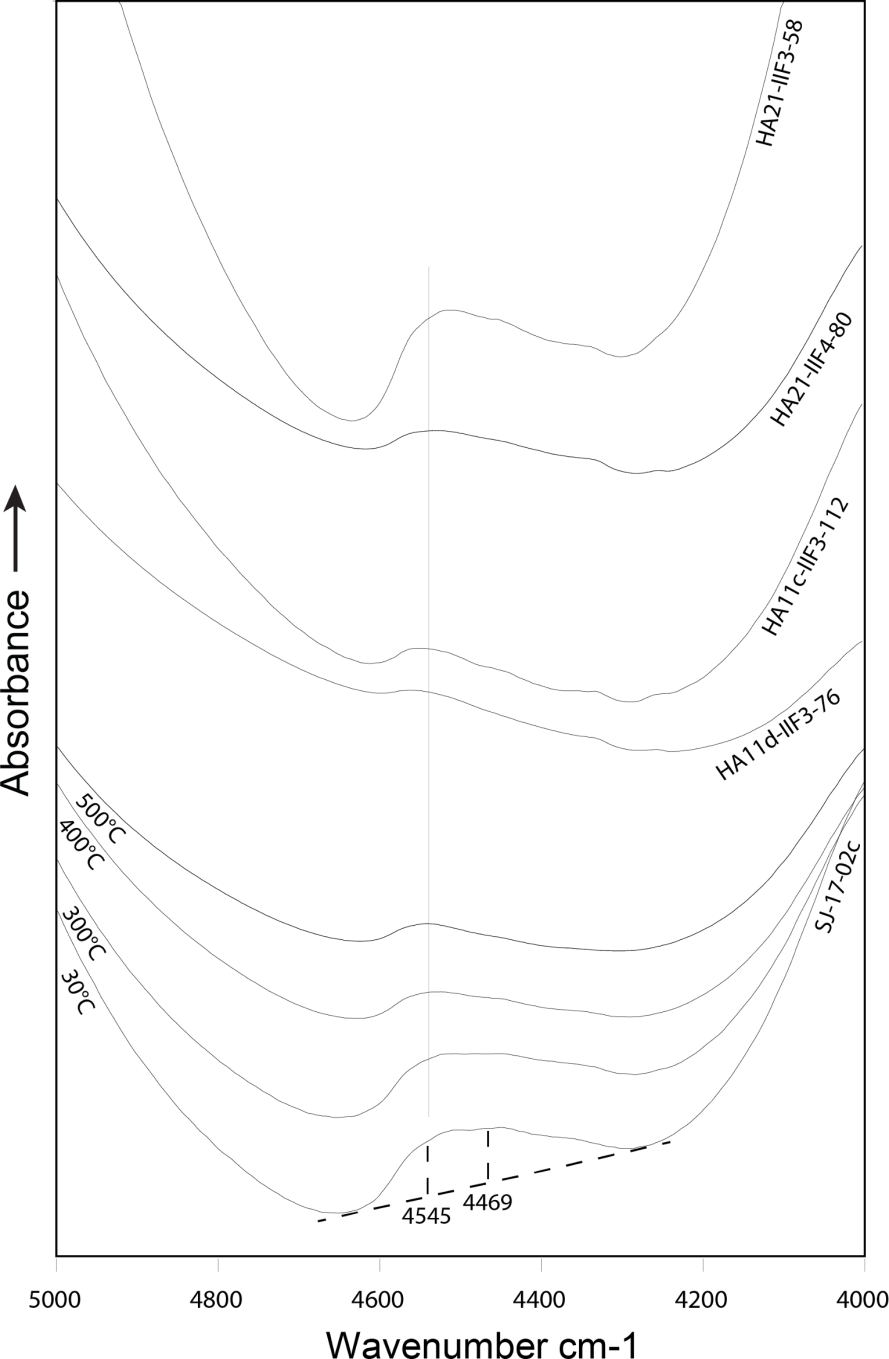
Near infrared transmission spectra between 5000 and 4000 cm^-1^. The measured spectral range contains a SiOH combination band. The baseline used for the measurement of the 4545/4469 cm^-1^ ratio are shown in broken lines. The four spectra in the lower part correspond to the experimental heating series of geological reference sample SJ-17-02c. Note the gradual shift of the band to higher wavenumbers with rising heating temperature (use the vertical line as visual guideline). The four spectra in the top were recorded from archaeological samples. The lower two archaeological samples HA11d-IIF3-76 and HA11c-IIF3-112 were assigned to the groups *Gloss contrast* and *Test* respectively. Both were found to be heated by their 4545/4469 cm^-1^ ratio. The uppermost two samples HA21-IIF4-80 and HA21-IIF3-58 were assigned to the group not-heated as confirmed by the 4545/4469 cm^-1^ ratio. Spectra are vertically offset for readability.

IR transmission was recorded at normal incidence using unpolarised light of a Bruker VERTEX 80v spectrometer, set up with a Near IR detector. Spectra were acquired between 4000 and 5000 cm^-1^ with a resolution of 8 cm^-1^. The IR light was directly transmitted through the samples fixed in the spectrometer’s sample chamber. The diameter of the IR beam was cut to 5mm by a circular diaphragm. No other sample preparation was necessary and the analyses of all archaeological samples remained non-destructive. These IR analyses were performed in collaboration with K.G. Nickel of the Department of Geosciences (Applied Mineralogy) of the University of Tübingen. The baseline used for the measurement of the 4545/4469 cm^-1^ ratio was a straight line between the lowest two points on either side of the SiOH absorption band (Fig 3). Experimental errors for artefacts and unheated geological reference samples correspond to the range of values measured on the 10 unheated geological samples and were set to a fixed value of ±0.04. This error is due to sample heterogeneities and reflects the inter- and intra-sample variability of Jurassic chert from the Swabian Alb. Error bars for the experimental heating series, used for temperature determination, correspond to the minimum and maximum extension of the values measured on the 11 artefacts in the *Not-heated* group and was set to a fixed value of ±0.08 (this is further explained in the results section). This error takes into account the sample heterogeneity of Jurassic chert but also reflects the range of different values produced from samples collected in the Helga-Abri archaeological deposits (these may also be influenced by taphonomic agents). We chose this larger error for the experimental series to increase the significance of the temperature determination for all analysed artefacts.

LSM measurements were performed using a Keyence VK-X 100 and a 20x objective. To obtain 3D surface models of the removal scars, four tiles were stitched together, producing 1.3 mm x 0.95 mm measuring images. These surface models were then corrected for surface inclination and the arithmetical mean roughness Ra in μm was measured from the entire surface (using the Gwyddion software). No additional filtering was applied and the produced Ra values must be considered a mixture between surface waviness and roughness. These analyses were conducted in collaboration with C. Berthold of the Competence Center Archaeometry—Baden-Wuerttemberg (CCA-BW) at Tübingen University’s Department of Geosciences.

## Results

### 4545/4469 cm^-1^ ratio values and heating temperatures

**Fig 4:**
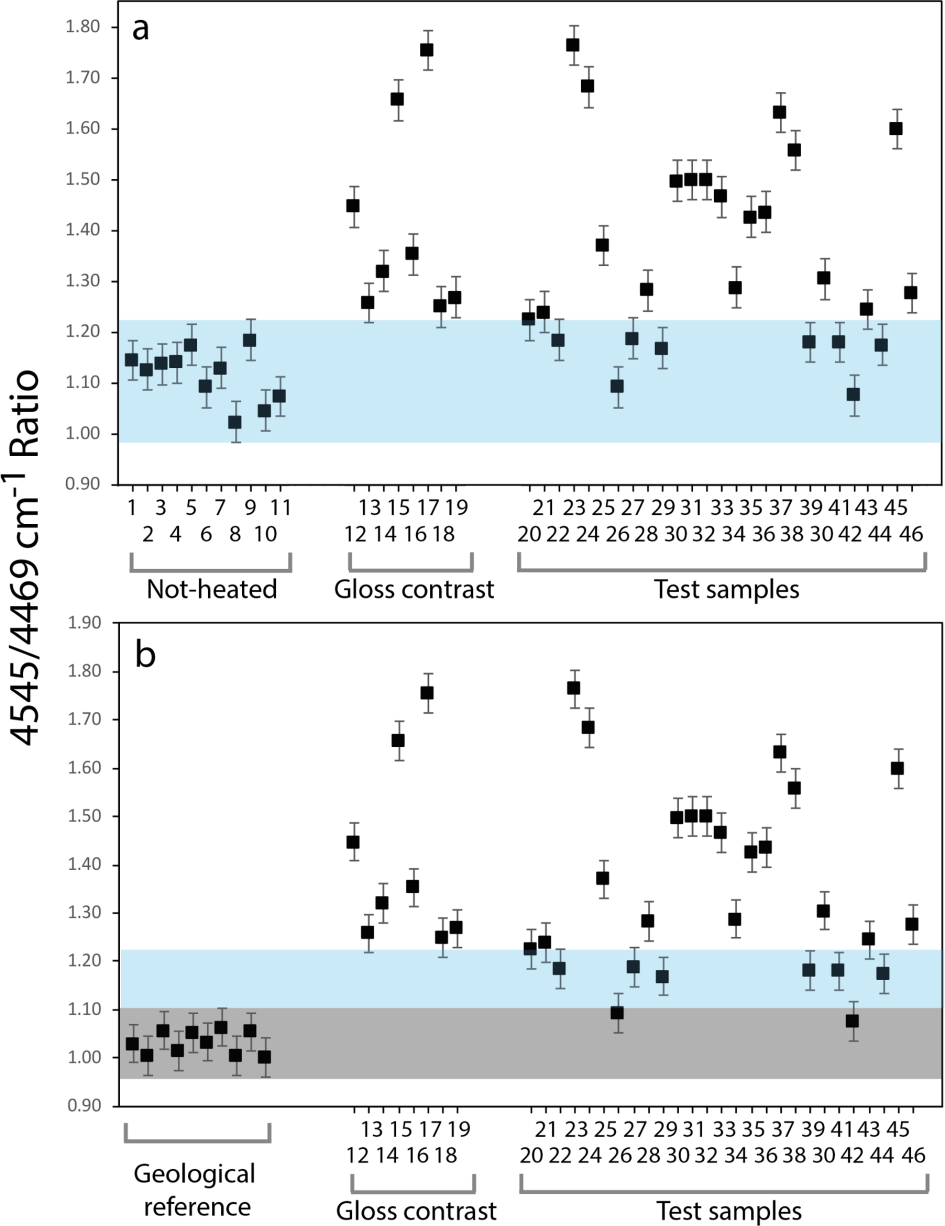
Plots of the values of the 4545/4469 cm^-1^ ratio obtained from Helga-Abri samples. Archaeological samples are named using the sample numbers shown in Table 1. a: Comparison between the ratios of the three archaeological groups. The range of values produced by samples of the group *Not-heated* is marked by a blue bar. All of the archaeological samples with gloss contrast and some from the *Test* group are clearly distinguished by their 4545/4469 cm^-1^ ratio value and can be identified as heat-treated (i.e. they plot above the blue bar) but some samples of the *Test* group produced values identical to the not-heated archaeological reference. b: Comparison between the ratios obtained from not-heated geological references samples with artefacts from the groups *Gloss contrast* and *Test*. The range of geological reference values is marked by a grey bar and the range of values obtained from artefacts of the group *Not-heated* is still marked by a blue bar. Note that *Not-heated* artefacts produced slightly higher values than the geological reference.

Fig 3 shows the spectra of the geological reference sample experimentally heat-treated to successive temperatures and four spectra of archaeological samples for comparison (a more detailed deconvolution of these SiOH bands can be seen in S1 Fig and S2 Fig). The SiOH band of the experimentally heated sample shifts progressively to higher wavenumbers with rising temperature. Thus, the 4545/4469 cm^-1^ ratio values deduced from the band allow to estimate the heating temperatures of the archaeological samples. These ratio values are listed in Table 2 for geological and experimental sample and in Table 3 for artefacts. Fig 4A is a plot of the ratio values of the 46 archaeological samples. Artefacts previously assigned to the group *Not-heated* have 4545/4469 cm^-1^ ratio values between 1.023 and 1.184, setting the range of ratio values of the unheated archaeological Jurassic chert to between ~0.983 and ~1.224 if the estimated error is taken into account (blue bar in Fig 4A). Archaeological samples previously assigned to the group *Gloss contrast* (i.e. samples showing an unambiguous proxy of knapping after heat treatment), all have higher ratio values than the *Not-heated* samples (between 1.249 and 1.754), confirming that they were heat-treated. The range of these values are most likely caused by different temperatures used for heating [20, 37]. Samples previously assigned to the *Test* group produced ratio values between 1.076 and 1.764, indicating that the group may contain both heat-treated and not-heated artefacts. Samples from the *Test* group that plot within the blue bar of Fig 4A may be considered as not-heated based on these results. This is the case of eight pieces. However, comparing the archaeological groups *Gloss contrast* and *Test* with geological reference samples (Fig 4B), a slightly different pattern can be observed. The grey bar in Fig 4B marks the scattering ranges of values obtained from geological reference samples (the upper part of the blue bar from Fig 4A is maintained here for comparability). Here, only two samples appear as clearly not-heated. However, it must be stressed that the difference between taphonomic processes acting upon archaeological *Not-heated* and geological samples is unknown and the significance of the comparison in Fig 4B cannot be evaluated. The only secure observation stemming from these results is that two of the test samples were most likely not-heated and six *Test* pieces remain indeterminate with respect to them being heated or not.

**Table 3:** Heat treatment parameters of artefacts.

Macroscopic Group	Short sample N°	Colour	Gloss Contrast (GC)	Overall gloss	Ra (μm)	Ra (μm) pre-HT of GC samples	IR Hydration Ratio
**Not-heated**	1	yellowish-grey	no	weak	4.80		1.144 ±0.04
**Not-heated**	2	grey	no	weak	8.69		1.126 ±0.04
**Not-heated**	3	yellowish-grey	no	weak	4.25		1.137 ±0.04
**Not-heated**	4	yellowish-brown	no	weak	4.09		1.141 ±0.04
**Not-heated**	5	yellowish-brown	no	weak	1.64		1.174 ±0.04
**Not-heated**	6	yellowish-grey	no	int.	2.09		1.092 ±0.04
**Not-heated**	7	yellowish-brown	no	weak	2.85		1.129 ±0.04
**Not-heated**	8	light yellowish-grey	no	weak	2.21		1.023 ±0.04
**Not-heated**	9	light yellowish-grey	no	weak	3.00		1.184 ±0.04
**Not-heated**	10	light yellowish-grey	no	int.	2.89		1.046 ±0.04
**Not-heated**	11	yellowish-grey	no	weak	2.93		1.073 ±0.04
**Gloss contrast**	12	yellowish-brown	yes	-		4.12	1.447 ±0.04
**Gloss contrast**	13	reddish-yellow	yes	-		3.47	1.258 ±0.04
**Gloss contrast**	14	light reddish-grey	yes	-	1.55		1.320 ±0.04
**Gloss contrast**	15	light reddish-grey	yes	-	1.69	7.24	1.656 ±0.04
**Gloss contrast**	16	reddish-grey	yes	-	2.33	4.45	1.353 ±0.04
**Gloss contrast**	17	grey	yes	-	2.51	6.61	1.754 ±0.04
**Gloss contrast**	18	reddish-grey	yes	-	3.48		1.249 ±0.04
**Gloss contrast**	19	light reddish-grey	yes	-	1.75	2.75	1.268 ±0.04
**Test**	20	reddish	no	int.			1.225 ±0.04
**Test**	21	grey	no	int.	1.80		1.239 ±0.04
**Test**	22	grey	no	int.	4.39		1.184 ±0.04
**Test**	23	reddish	no	strong			1.764 ±0.04
**Test**	24	light yellowish-grey	no	int.			1.683 ±0.04
**Test**	25	reddish	no	int.	2.20		1.370 ±0.04
**Test**	26	light reddish-brown	no	strong	2.06		1.093 ±0.04
**Test**	27	grey	no	strong			1.187 ±0.04
**Test**	28	light reddish-grey	no	strong	2.34		1.282 ±0.04
**Test**	29	grey	no	int.	3.17		1.169 ±0.04
**Test**	30	grey	no	int.	4.01		1.497 ±0.04
**Test**	31	reddish-grey	no	strong	2.06		1.500 ±0.04
**Test**	32	grey	no	strong	2.07		1.500 ±0.04
**Test**	33	grey	no	strong	1.97		1.466 ±0.04
**Test**	34	grey	no	strong	3.54		1.288 ±0.04
**Test**	35	dark grey	no	strong	2.49		1.426 ±0.04
**Test**	36	light reddish-grey	no	strong	1.46		1.436 ±0.04
**Test**	37	light reddish-grey	no	strong	1.36		1.632 ±0.04
**Test**	38	reddish	no	int.	2.67		1.558 ±0.04
**Test**	39	grey	no	strong	1.61		1.180 ±0.04
**Test**	40	grey	no	strong	2.15		1.305 ±0.04
**Test**	41	reddish-grey	no	int.	4.54		1.180 ±0.04
**Test**	42	yellowish-grey	no	int.	3.02		1.076 ±0.04
**Test**	43	reddish-yellow	no	int.	1.98		1.244 ±0.04
**Test**	44	dark yellow	no	strong	2.99		1.174 ±0.04
**Test**	45	yellowish-brown	no	int.	4.21		1.600 ±0.04
**Test**	46	reddish-brown	no	int.	3.63		1.277 ±0.04

Colour estimations are made visually and are meant as indications only. Overall gloss was only estimated when no gloss contrast was observed. Roughness values in the column ‘Ra (μm)’ are taken on the ventral surface of the artefacts when possible. Values in the column ‘Ra (μm) pre-HT of GC samples’ (GC = Gloss contrast) are taken on the identified pre-heating removal scars of artefacts in the group ‘Gloss contrast’.

The scattering of the *Not-heated* artefacts’ ratio values in Fig 4A indicates that the error of ±0.04, as determined from geological reference samples, may be too low when applied to the Helga-Abri artefacts: the mean of all *Not-heated* ratio values is 1.115; their maximum is 1.184, i.e. it lies 0.069 above the mean; values lower than the mean plot till -0.093 below the mean. However, the ±0.04 error bars are still maintained here for archaeological samples because the scattering of artefacts in the group *Not-heated* may alternatively be caused by some of these pieces having been subject to low-temperature heating (i.e. our initial assignment to the group *Not-heated* may be wrong). As this uncertainty cannot be cleared up in this study, we adjusted the error bars for the following temperature calibration, setting them to the scattering range of values obtained from the artefacts in the *Not-heated* group (±0.08). In this way, the resulting temperature estimations takes into account the uncertainty of our assignment but also the possibility that inter-sample heterogeneity of Jurassic chert used in Helga-Abri was larger than the one found in our three geological reference samples.

**Fig 5:**
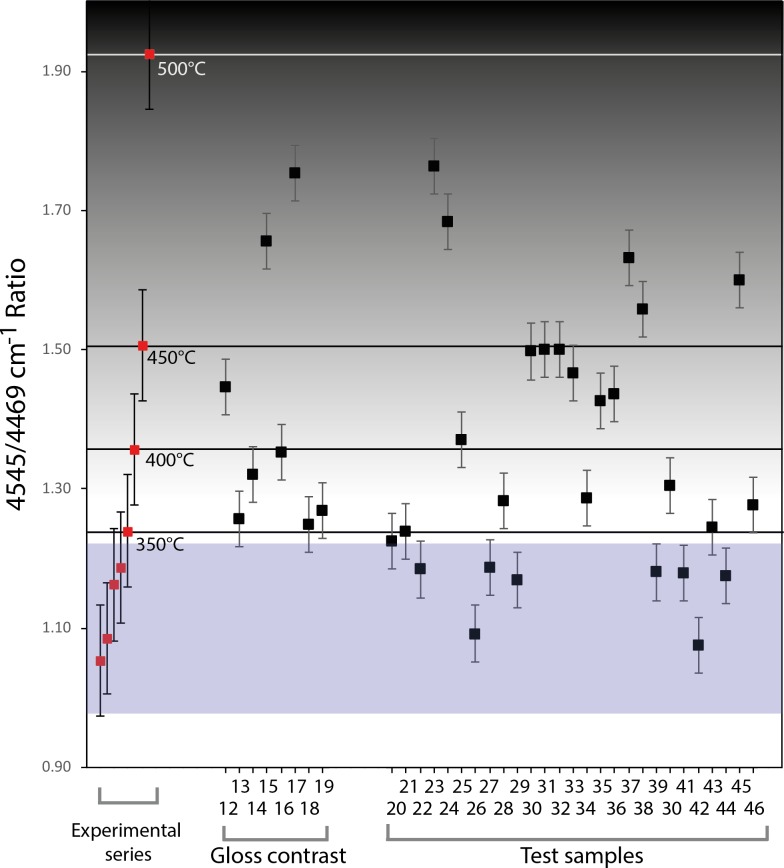
Comparison between the 4545/4469 cm^-1^ ratio values of archaeological and experimental samples for heating temperature estimation. Ratio values of the progressively heated geological reference samples are displayed on the left of the graph. Temperatures, as calibrated by this experimental series, are shown as horizontal lines. The blue bar is maintained from Fig 4A and marks the range of values produced by *Not-heated* archaeological samples. Artefacts plotting in this bar must be considered not-heated or heated to temperatures below 305°C. The experimental temperature calibration allows to estimate the heating temperature of most of the archaeological samples between 350°C and 500°C.

Fig 5 is a plot comparing the archaeological groups *Gloss contrast* and *Test* with experimentally heat-treated reference sample. Ratio values of the experimental series are gradually increasing after each temperature step. Only from 350°C upwards, these ratio values plot above the scattering range of the *Not-heated* archaeological samples (the blue bar from Fig 4A is maintained for comparability). Experimental ratio values below 350°C are statistically indistinguishable from *Not-heated* artefacts, further strengthening the interpretation that at least six *Test* samples must remain indeterminate (i.e. they may be unheated or heated to temperatures below 350°C). The experimental series allows however to estimate the heating temperatures of all artefacts in the group *Gloss contrast* and 19 samples from the *Test* group. These artefacts were heat-treated with temperatures between 350°C and 500°C.

### Ra values of Helga-Abri samples

**Fig 6:**
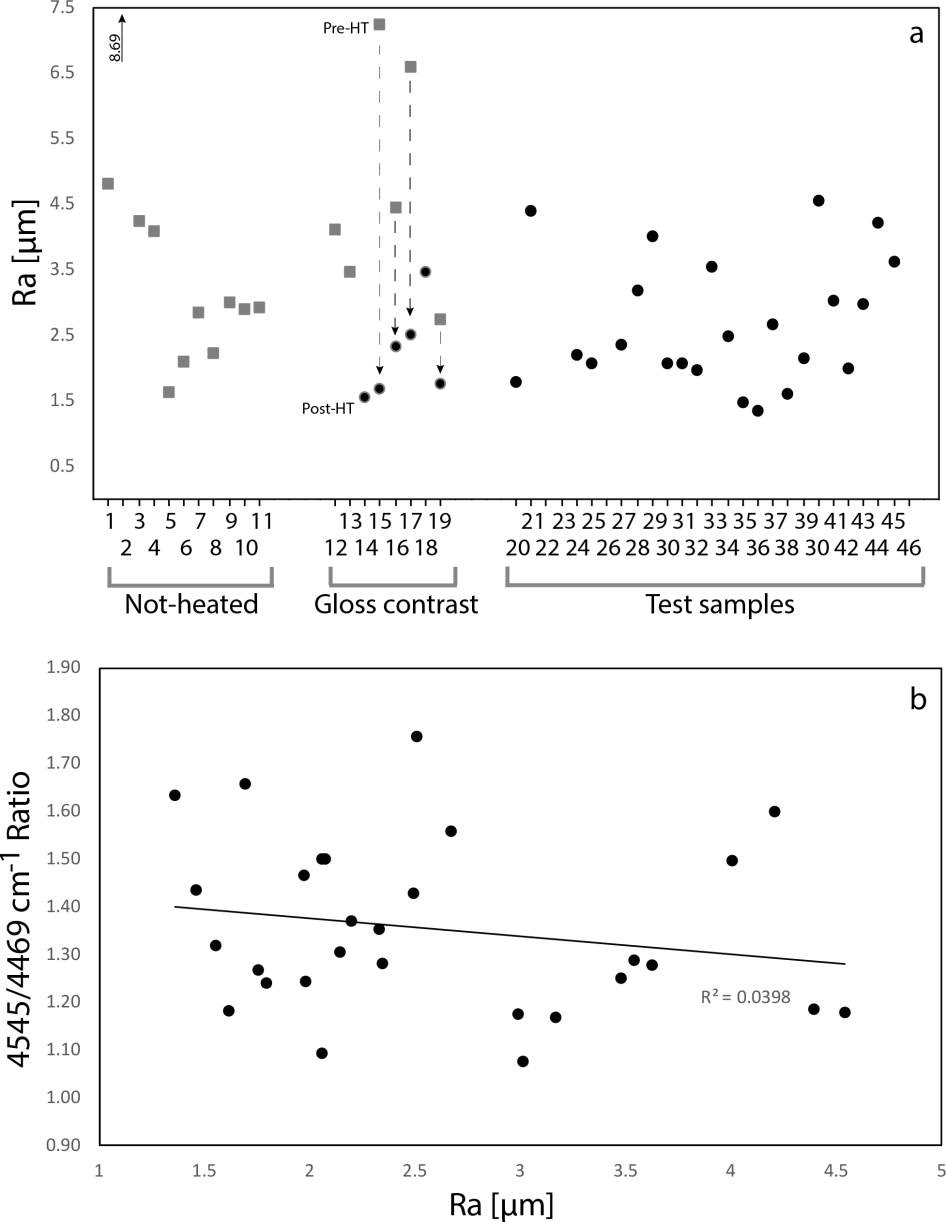
Plots of the mean roughness values (Ra) of archaeological samples. a: Plot of Ra values, maintaining the same arrangement of samples as in Fig 4. Note that the surface roughness values of *Not-heated* artefacts plot in the same range as *Test* samples. Two values are plotted for some samples of the *Gloss contrast* group. In this case, square dots are pre-heating removal scars and round dots are post-heating scars. Note that for all cases, where these double measurements were possible, post-heating scars are smoother than pre-heating scars. b: Correlation graph plotting the samples’ 4545/4469 cm^-1^ ratio value over their Ra value. Note the absence of correlation between surface roughness and heated vs. not-heated or heating temperature.

Fig 6A is a plot of the 42 archaeological samples’ Ra values. Ra values measured on the surfaces of samples previously assigned to the group *Not-heated* plot between 1.64 μm and 4.8 μm, with one apparent outlier plotting at 8.69 μm. The range of Ra values obtained on pre-heating removal scars of the group *Gloss contrast* roughly fall within this range. In all four cases where it was possible to measure pre- and post-heating scars on single *Gloss contrast* artefacts, post-heating scars have lower Ra values than pre-heating scars (as indicated by the broken arrows in Fig 6A). The range of Ra values measured on samples of the *Test* group is reasonably close (1.36–4.54 μm) to the range of values obtained from Not-heated artefacts. Because, at least, 19 of the *Test* samples were estimated to be heat-treated by IR analysis, surface roughness appears to be not significant for identifying heat treatment on Jurassic chert from the Swabian Alb. This observation is further strengthened by the correlation graph in Fig 6B. The estimated heating temperature, or even the identification of heat treatment or the absence of it on artefacts, stands in no statistical relation of dependence with the Ra values on the artefacts’ surfaces (as indicated by a coefficient of determination r^2^ of 0.0398). Thus the absence of a clear correlation between the 4545/4469 cm^-1^ ratio value and the sample’ surface roughness implies that roughness or gloss on the samples is not a good criterion to estimate heating temperature for this type of chert.

## Discussion

### The technique and parameters used for heat treatment at Helga-Abri

None of the analysed archaeological samples show any trace of overheating, so that the estimated heating temperatures of up to 500°C are most likely caused by intentional heat treatment and not post-depositional burning. Our IR analyses confirm that all archaeological samples with gloss contrast (i.e. unambiguous diagnostic heat-treated pieces) were indeed heat-treated, implying that Schmidt et al.’s [20] method works for the Jurassic chert used at Helga-Abri. Six *Test* samples appear to have been either not-heated al all, or heated to temperatures below 350°C, i.e. they remain indeterminate after our analysis, and two artefacts of the *Test* group were most likely not heated. Heating temperatures of the remaining, heat-treated, *Test* group artefacts do not appear to be well standardised, roughly falling between 350°C and 500°C. The range of 4545/4469 cm^-1^ ratio values, recorded on the artefacts, that led to estimate this temperature range is most likely caused by different temperatures of the heat treatment of each piece and not different heating durations, as reactions kinetics of the heat-induced processes taking place in chert were shown to be very fast [38]. Temperatures between 350°C and 500°C lie significantly above the temperatures determined for other archaeological assemblages that have yielded evidence of heat treatment. For example, heat treatment in the Neolithic Chassey culture (4200–3500 Cal BC) of southern France was a well calibrated process that produced temperatures between 200°C and 250°C in chert [20, 35]. Heat treatment in the European Upper Palaeolithic Solutrean (22000–17000 Cal BP) was a process that aimed at producing temperatures between 250°C and 300°C in chert [37]. In order to produce, control and maintain such temperatures, a specific heating environment or oven-like structure must be built. For example, sand-baths or similar underground heating structures allow to heat-treat stone with a range of temperatures from 200°C to 400°C [19, 39, 41, 42], allowing for good control of the desired temperature range by choosing specific fire woods and modifying the nature/thickness of the insulating sediment. However, temperatures recorded on Jurassic chert from Helga-Abri are significantly higher than the ones recorded from Chassey and Solutrean artefacts. How can these differences be explained? Many of the known chert varieties have ideal heating temperatures between 200 and 350°C [25, 43, 44]. Most of these samples become even less well suited for stone knapping after heating above these temperatures, normally not withstanding heat treatment as high as 500°C (see for example [45, 46]). The reason of this is their content of molecular/chemically-bound water and the pore-space available for ‘water’ evacuation [30]. Most types of chert contain ‘water’ of up to 1.5 wt% [33, 47, 48] and an intergranular pore-spaces of 0.5–1.5 vol% [25, 33, 34, 49, 50], imposing an upper limit of ~350°C (and exceptionally 450°C in very small samples) for heat treatment [25, 33, 44]. The upper limit of heating temperatures of Helga-Abri chert, close to 500°C, was thus only made possible by an exceptional thermal stability of this type of chert. No explicit data on the ‘water’ content/pore-space of Jurassic chert are available to date, but our observation that none of the experimental samples showed any sign of overheating after heating to 500°C suggests that these rocks have low ‘water’ content and/or large pore-space (i.e. good resistance against thermal fracturing up to high temperatures). This hypothesis is further strengthened by an earlier study on heat treatment in the Mesolithic of southwestern Germany [19] that found that “…Jurassic cherts are usually very heat resistant.” (p. 328).

The degree of standardisation allowed by the technique used at Helga-Abri also seems to be significantly lower than in other periods. Temperature ranges of ±25°C in the Neolithic Chassey [20] and ±~30°C in the Upper Palaeolithic Solutrean [37] are significantly narrower than in the Mesolithic of Helga-Abri (±75°C). Standardised heating techniques, such as sand-baths or earth-ovens, are unlikely to produce such great scattering ranges of heating temperatures, precluding the hypothesis of their use in the Mesolithic of southwestern Germany. There is, however, one example of stone heat treatment that may be compared with our data in terms of heating parameters: the earliest known examples of heat treatment, dating to the southern African Middle Stone Age, where silcrete, a silica rock significantly coarser than chert, was heated [41, 51]. These silcretes were heat-treated at temperatures close to 400°C, using the above-ground part of burning wood fires [52, 53]. Depending on the used wood, temperatures as high as 550°C can be attained with this technique [53]. Such high temperatures, and the fast heating speeds associated with this heating technique, are withstood by silcrete because it contains a significantly lower amount of ‘water’ and larger pore-space than chert [43, 54]. Using open-air fires for heat treatment can be expected to produce a wider range of heating temperatures when the stones are placed at different parts of the embers or ashes. Depending on the quantity of embers in contact with the stones and their local temperature or degree of cooling, both being quantities that are highly variable and difficult to control in open fires (see for example [55]), stones heat-treated with this technique would experience a wide range of actual temperatures. The pattern observed in Helga-Abri artefacts (relatively high temperature; low standardisation) can thus be reasonably well explained by the hypothesis that Jurassic chert was heat-treated in the above-ground part of camp-fires like structures.

### An isolated phenomenon in the Mesolithic of southwestern Germany?

Are there other Early Mesolithic assemblages in Europe that can be compared with the pattern we observe at Helga-Abri? Several mentions of heat treatment can be found in the current literature on the northern European Mesolithic. For example, a Mesolithic assemblage from the Netherlands, contemporary to our Helga-Abri assemblage, was analysed for potential chert heat treatment [56] but the authors concluded that intentional heating was not part of the reduction sequence at the site; the observed pattern most likely resulted from accidental burning. Another work [57] tested for heat treatment in the Mesolithic of southern Sweden but also concluded that none of the analysed artefacts were heat-treated. Yet another work [58] claims that intentional heat treatment was practised in the Mesolithic Janisławic culture (6600–4600 Cal BC) of Poland, but the literature cited within this work contains either no comment on heat treatment at all, or only short allusions to its presence ([59–61] cite in [58]). There are other descriptions of chert “probably” being heat-treated in the Mesolithic Butovo culture (8000–5000 BC) of the Russian Volga basin [62, 63] but no detailed descriptions of specific artefacts, that would allow to evaluate its presence with certainty, are available. Thus, heat treatment in northern European Early Mesolithic cultures is either dismissed or only shortly mentioned in the current literature. No detailed studies or descriptions of artefacts with unambiguous proxies used for recognising intentional heating, like gloss contrast, are available. In light of the available but incomplete literature sources, the only conclusion we can draw is that intentional heat treatment may have been practised further north than the Beuronian, yet no qualitative or quantitative comparisons can be made to date. There is however one other Early Mesolithic context in southern Europe that can be compared with our Helga-Abri data. Intentional pyrofracturation, for the reduction of nodule size and the creation of new angles for knapping, was described in the Sauveterrian (8500–7000 Cal BC) of the French Vaucluse region [64, 65]. High temperatures and/or fast heating rates, as they are typically produced in open fires, are necessary for flint to fracture [30]. Slow and low-temperature heating conditions are not efficient to produce heat-induced fracturing in silica rocks ([30, 66], for silica rocks other than chert see [67]). A testable hypothesised may therefore be that heat treatment in the French Sauveterrian relied on a similar technique as at Helga-Abri (i.e. the use of open fires) and that such a heating technique would be characteristic for the central and southern European Early Mesolithic. Only spatially more extensive future works can shed light on the probability of this hypothesis.

### Recognising heat treatment on Jurassic chert

Another results of this study is the apparent difficulty to identify heat-treated artefacts in Mesolithic assemblages made from Jurassic chert. Colour alteration has in the past been used to identify heat-treated artefacts in the Beuronian of the Swabian Alb (see for example [12]). The reasoning behind this was that Jurassic chert contains iron-rich inclusions that unequivocally turn red upon heating. However, as can be seen from Table 3, not all heat-treated artefacts from Helga-Abri are red. Some remained grey although they were heated. Our experimentally heat-treated geological sample also did not turn red but remained grey. These observations lead to the interpretation that iron-rich inclusions are not ubiquitous in Jurassic chert. Colour-based identifications of heat treatment within assemblages made from such rocks would therefore potentially exclude a significant part of the artefacts from the determination (the ones containing little or no iron). The only secure proxy in this regard appears to be that if Jurassic chert artefacts are bright yellow (i.e. contain untransformed iron-rich impurities), they were not heated.

Another often used heating proxy is the overall gloss on artefacts. The reflectivity of removal scars, commonly described as surface lustre, shine or gloss is partly controlled by surface roughness. Thus, the phenomenon actually observed when estimating surface gloss is surface roughness. The shininess of the removal scars on chert artefacts (often used relatively, comparing the gloss on different artefacts from within an assemblage; see for example [68, 69]) has in the past been used successfully to determine whether they were knapped before or after heat treatment. Classifying artefacts in categories (strong vs. weak overall gloss), it may be possible to estimate the relative percentages of heated artefacts in an assemblage [18]. However, Ra values measured on the post-heating removal negatives of heat-treated Jurassic chert showed a range of values similar to the Ra values measured on unheated samples, hence, not allowing to distinguish between heat-treated and unheated Jurassic chert. Our data therefore strongly indicate that overall gloss cannot reliably be used as heating proxy on this type of chert. The inter-sample variability, or heterogeneity, simply seems to be too great in Jurassic chert, different varieties producing different surface roughness values even without heat treatment, so that the difference between rougher pre- and smoother post-heating scars is overlapped by it. Gloss contrast directly observed on a single piece however, appears to be a good and unambiguous proxy because all four analysed *Gloss contrast* samples showed post-heating removals that are smoother than their pre-heating removals.

This finding, that neither colour, nor overall surface gloss can be unambiguously used to identify heat treatment in the Swabian Alb is rather inconvenient for Mesolithic archaeologists. There seems to be no macroscopic proxy that allows to identify heat treatment in the absence of gloss contrast. Only such pieces with gloss contrast can be regarded as diagnostic and used to positively identify heat treatment in assemblages made from Jurassic chert. This creates the inherent disadvantage that quantitative estimations of the number of heat-treated artefacts in such assemblages cannot rely on macroscopic criteria. Non-destructive archaeometric techniques, as the one used in this work, may however help to approach this issue.

## Conclusion and implications

The results in this paper allow to put lithic heat treatment in the Early Mesolithic of southwestern Germany into perspective. As Eriksen [18] already highlighted in an earlier work, a low-investment, cost- and time-effective heating technique, relying on the active part of above-ground fires, would somehow mirror the simplification occurring in the Beuronian lithic reduction sequence with respect to older periods, a sequence that sometimes has even been described as opportunistic [14]. Based on her experimental work [19], Eriksen hypothesised that such a low-investment technique may have been used in the Beuronian of the Swabian Alb. Our results support her hypothesis. Heat treatment was practised at high temperatures, probably using regular camp fire structures, and heating parameters were not well controlled. A structure that may potentially have been used for above-ground heating, a stone-lined fire place, was found in the Helga-Abri deposits [21], further strengthening the hypothesis of opportunistic heat treatment.

This pattern may spread beyond the Swabian Alb as some examples of pyrofracturation, coming from the French Sauveterrian, can be interpreted to be conducted with similar technical processes. Heating stone in open fires might even be a hitherto unrecognised trait of southern and central European Early Mesolithic cultures. The spatially and temporally next closest similar heating technique, relying on open-air fires, can only be found in the South African Later Stone Age [70], a far-away and entirely unrelated context. Is this a case of technological convergence in the archaeology of heat treatment? Is the use of specific heating techniques in different archaeological contexts (e.g. sand bath; earth oven; open-air fires) the result of pure coincidence? Or are heating techniques imposed by the used materials, their thermal stability and the heating parameters they require? Only future studies, taking into account a larger set of contexts having yielded traces of heat treatment, may shed light on these questions. A thorough mineralogical study of the Helga-Abri artefacts’ raw material would allow to better understand the apparent thermal stability of Jurassic chert. It would also provide better insight into the choices Mesolithic hunter-gatherers made with respect to specific raw materials. Unfortunately, destructive petrographic analyses lied outside of the scope of this first study but it is planned to conduct such analyses on another, larger, assemblage from another site in the Swabian Alb region, to further investigate Mesolithic heat treatment.

Another important implication of this study is the finding that many of the macroscopic proxies normally used to identify heat treatment in archaeological assemblages seem not to work in the context of the Swabian Alb. Estimating overall gloss on undiagnostic pieces (i.e. pieces without gloss contrast) does not allow to count heat-treated vs. not-heated pieces in assemblages made from Jurassic chert. The same is true for colour. This result, inconvenient as it is, must be taken into account in (re-)interpreting existing works on Jurassic chert that used macroscopic estimations of heat treatment. Future works should rely on a combination of traditional archaeological methods and non-destructive archaeometric techniques.

## Supporting information

S1 Fig Band components obtained by fitting of reference sample spectra. Sample SJ-17-02c, lower spectrum unheated and upper spectrum experimentally heat-treated with 550°C. The three components lie at 4545, 4460 and 4359 cm^-1^. The Root Mean Squared Error (RMSE) of the fit of the unheated reference spectrum (lower spectrum) is 0.00013 and the RMSE of the (upper) spectrum of the heated reference is 0.000286. The comparison shows that spectra of both heated and unheated samples can be reasonable well fitted with three components at identical wavenumbers. Only their relative high changes.
(TIF)

S2 Fig Band components obtained by fitting of archaeological spectra. The lower spectrum belongs to a sample than was found to be not-heated by its 4545/4469 cm^-1^ ratio (HA11d-II-F3-76), the upper spectrum to a sample than was found to be heat-treated (HA21-II-F3-58). The three components lie at the same wavenumbers as in S1 Fig. Again, only their relative height changes. Note the supplementary two bands at low wavenumbers that are not present in the spectra of reference samples. They might be due to residues of the coating or pen used to label the pieces or to other unknown factors that result from their conditioning in the collection facility they are curated in. In any case, their presence does not inflict upon the quality of the measurements that lead to calculate the 4545/4469 cm^-1^ ratio.
(TIF)
