# Supplementary figures and images for: Stone heat treatment in the Early Mesolithic of southwestern Germany: Interpretation and identification
Source: PLoS One. 2017 Dec 6;12(12):e0188576. doi: 10.1371/journal.pone.0188576 (PMC5718480; doi:10.1371/journal.pone.0188576)

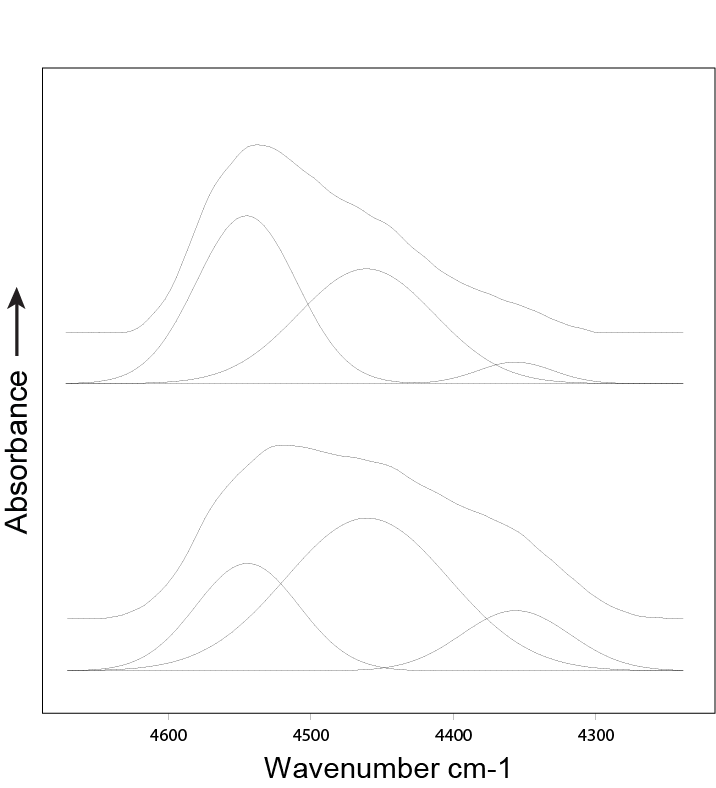

Supplement: S1 Fig — Sample SJ-17-02c, lower spectrum unheated and upper spectrum experimentally heat-treated with 550°C. The three components lie at 4545, 4460 and 4359 cm-1. The Root Mean Squared Error (RMSE) of the fit of the unheated reference spectrum (lower spectrum) is 0.00013 and the RMSE of the (upper) spectrum of the heated reference is 0.000286. The comparison shows that spectra of both heated and unheated samples can be reasonable well fitted with three components at identical wavenumbers. Only their relative high changes. (TIF) [file pone.0188576.s001.tif]

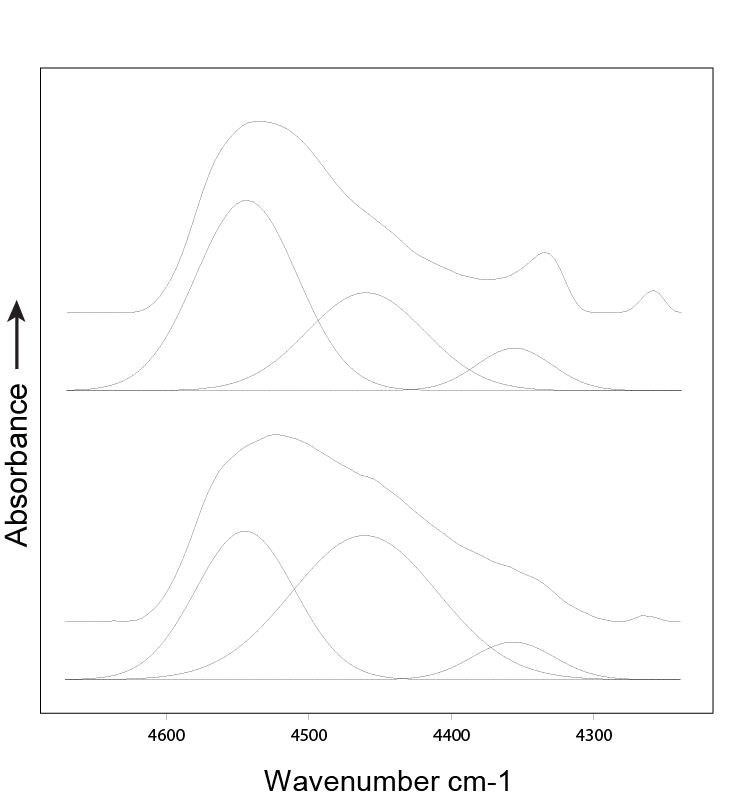

Supplement: S2 Fig — The lower spectrum belongs to a sample than was found to be not-heated by its 4545/4469 cm-1 ratio (HA11d-II-F3-76), the upper spectrum to a sample than was found to be heat-treated (HA21-II-F3-58). The three components lie at the same wavenumbers as in S1 Fig. Again, only their relative height changes. Note the supplementary two bands at low wavenumbers that are not present in the spectra of reference samples. They might be due to residues of the coating or pen used to label the pieces or to other unknown factors that result from their conditioning in the collection facility they are curated in. In any case, their presence does not inflict upon the quality of the measurements that lead to calculate the 4545/4469 cm-1 ratio. (TIF) [file pone.0188576.s002.tif]
